# Relationship between sensory-processing sensitivity and age in a large cross-sectional Japanese sample

**DOI:** 10.1016/j.heliyon.2019.e02508

**Published:** 2019-10-03

**Authors:** Yuki Ueno, Aki Takahashi, Atsushi Oshio

**Affiliations:** a Center for Evolutionary Cognitive Sciences, Graduate School of Art and Sciences, The University of Tokyo, 3-8-1 Komaba, Meguro-ku, Tokyo, 153-8902, Japan; b Faculty of Psychology, Chukyo University, 101-2 Yagoto Honmachi, Showa-ku, Nagoya-shi, Aichi, 466-8666, Japan; c Faculty of Letters, Arts and Sciences, Waseda University, 1-24-1 Toyama, Shinjuku, Tokyo, 162-8644, Japan

**Keywords:** Neuroscience, Sensory-processing sensitivity, Highly sensitive person, Age, Large cross-sectional study, Japanese adults

## Abstract

Sensory-processing sensitivity is a trait involving inherent individual differences that typically manifest in the brain's handling of sensory information (Aron and Aron, 1997). Studies regarding sensory-processing sensitivity have focused on specific age ranges; however, developmental changes in sensory-processing sensitivity have not been studied. This study aimed to examine the relationship between sensory-processing sensitivity and age in Japanese adults (*N* = 1,983, 1,078 men). Participants ranging in age from 20-69 completed the Japanese version of the 19-item Highly Sensitive Person Scale (Takahashi, 2016). Results of hierarchical multiple regression analysis indicated that low sensory threshold and ease of excitation decrease linearly with age, whereas aesthetic sensitivity increases linearly with age. In contrast, age-related changes in sensory-processing sensitivity do not differ by sex. Thus, the status of age-related changes differs slightly based on sensory-processing sensitivity factors.

## 1 Introduction

People experience and respond differently to various stimuli every day, including loud sounds, strong smells, and bright lights (Aron, 1997). The concept of sensory-processing sensitivity (SPS) represents individual differences in somatic sensation. SPS is an inherent individual difference that typically manifests in the brain's handling of sensory information (Aron and Aron, 1997; Aron et al., 2012). SPS has been reported as an important survival strategy (Kagan, 1994). People with high SPS are known as highly sensitive persons (HSPs) and account for 15–20% of the total population (Aron and Aron, 1997). Characteristics of HSPs include sensitivity to subtle stimuli and the tendency to experience a state of hyperstimulation. They are also more prone to depression and anxiety (e.g., Aron, 2010; Aron and Aron, 1997; Takahashi, 2016), while also scoring higher in empathy, justice, ethics, and sensitivity to beauty and art (e.g., Aron, 2010).

SPS is positively associated with neuroticism, and HSPs tend to experience hyperarousal and become more emotional under stress (Aron and Aron, 1997). However, SPS and neuroticism only have a middle-level magnitude of correlation (Aron and Aron, 1997; Smolewska et al., 2006). Aron and Aron (1997) also indicated that HSPs differ from neuroticism. Approximately 30% of HSPs are extroverts, and are known to be highly sensitive to beauty, art, and music, and have a rich sense of imagination (Aron and Aron, 1997).

Studies regarding SPS have focused on specific age ranges; developmental changes have not been studied. A study by Soto et al. (2011), involving 1,267,218 participants from English-speaking countries, revealed that neuroticism generally decreases with age, especially after adulthood. In a study of Japanese adults (*N* = 4,588), Kawamoto et al. (2015) showed a negative linear effect of age on neuroticism; it is reasonable to predict that SPS also changes with age. Caspi, Roberts, and Shiner (2005) argued for the relevance of the mutuality principle, which states that personality traits develop in a socially desirable fashion after adulthood.

Additionally, aging effects may differ depending on the three dimensions of SPS: low sensory threshold, ease of excitation, and aesthetic sensitivity (Smolewska et al., 2006; Takahashi, 2016). Low sensory threshold and ease of excitation, which are aspects of negative affect, are more highly associated with neuroticism than others (Smolewska et al., 2006). Therefore, scores should decrease linearly with age, similar to the age-related changes of neuroticism. Aesthetic sensitivity, an aspect of orienting sensitivity related to spirituality, is more highly correlated with openness than neuroticism (Smolewska et al., 2006). Openness tends to increase linearly with age, although it may decrease after middle age on account of the negative curve effect (Lehmann et al., 2013). According to Soto et al. (2011), openness increases only slightly after adulthood. Taken together, aesthetic sensitivity should increase with age, similar to openness.

The present study examined age-related changes in the three dimensions of SPS in Japanese adults aged 20–69. Previous studies suggest that age-related changes especially differ depending on the three dimensions of SPS (e.g., Smolewska et al., 2006; Takahashi, 2016). Although developmental changes should ideally be examined through long-term longitudinal studies, those related to personality traits can be investigated by comparing age groups using a large sample size in a cross-sectional survey. Previous studies have also reported the effects of sex on SPS (e.g., Aron and Aron, 1997; Benham, 2006; Takahashi, 2016). Drawing from existing studies (Kawamoto et al., 2015; Lehman et al., 2013), this research analyzed the effects of age on SPS using multiple regression models considering interactions between sex and age from the perspective of primary (linear relationships) and secondary (curvilinear relationships) effects.

## 2 Materials and methods

### 2.1 Participants and procedures

The survey was outsourced to an online survey company (Cross Marketing Co., Ltd.) and conducted using the survey software Qualtrics in January 2017. The respondents were Japanese residents from a wide range of age groups and regions who provided their consent to participate. Cross Marketing awarded reward points for the completion of the questionnaire. Respondents who violated the instructional manipulation check (Oppenheimer et al., 2009) were also excluded. Data of Japanese adults (*N* = 1,983, 1078 men; mean age = 48.85 years, *SD* = 10.87) from 47 prefectures, aged 20–69, were analyzed. The survey was conducted anonymously and in accordance with Cross Marketing Co. Ltd.’s personal information processing policy. Participation was entirely voluntary. Before the administration of the questionnaire, the participants were informed about the survey overview and terms of confidentiality, and their informed written consent was obtained. However, we did not explain to the participants the relationship between SPS and age in the aims of the present study. The survey was approved by the institutional ethics committee at which the author was affiliated.

### 2.2 Measures

The questionnaire included items on personal attributes, such as sex and age, and the Japanese version of the 19-item Highly Sensitive Person Scale (HSPS-J19; Takahashi, 2016), which is a translation of the 1997 version of the HSPS (Aron and Aron, 1997). The HSPS-J19 comprises three dimensions: low sensory threshold (e.g., “Are you bothered by intense and chaotic stimuli, such as loud noises?“), ease of excitation (e.g., “Do you get rattled when you have a lot to do in a short amount of time?“), and aesthetic sensitivity (e.g., “Do you notice and enjoy delicate or fine scents, tastes, sounds, and works of art?“). The HSPS-J19 has been tested for validity and reliability (Takahashi, 2016). The HSPS (Aron and Aron, 1997) consists of one factor and 27 items, whereas the HSPS-J19 excludes eight items with particularly low factor loading. Although the Japanese version does not contain the same number of items as the scale developed by Smolewska et al. (2006), their factor structures are identical. In the present study, the participants responded using a seven-point scale ranging from “strongly disagree” to “strongly agree.” Cronbach’s *α*, which indicates a scale’s internal consistency, was found to be .831 (95% CI [.820, .842]) in low sensory threshold, .808 (95% CI [.795, .820]) in ease of excitation, and .620 (95% CI [.592, .647]) in aesthetic sensitivity. Following Lionetti et al. (2018), Smolewska et al. (2006) and Takahashi (2016), a confirmatory factor analysis (one-factor model, two-factor model, and three-factor model) and bifactor model was conducted assuming a bifactor model structure. The fit indices of the bifactor model were as follows: *χ*^2^ = 1263.639, *df* = 133, *p* < .001, GFI = .935, AGFI = .907, CFI = .913, RMSEA = .065 (90% CI [.062, .069]), AIC = 1377.639. They were better than the fit indices of the one-factor model (*χ*^2^ = 3169.404, *df* = 152, *p* < .001, GFI = .839, AGFI = .798, CFI = .769, RMSEA = .100 (90% CI [.097, .103]), AIC = 3245.404), the two-factor model (*χ*^2^ = 2437.680, *df* = 151, *p* < .001, GFI = .870, AGFI = .837, CFI = .825, RMSEA = .087 (90% CI [.084, .090]), AIC = 2515.680), and the three-factor model (*χ*^2^ = 2315.862, *df* = 149, *p* < .001, GFI = .873, AGFI = .838, CFI = .834, RMSEA = .086 (90% CI [.083, .089]), AIC = 2397.862).

## 3 Results

HAD 16.012 (Shimizu, 2016), IBM SPSS Statistics Ver. 20.0, and IBM Amos Ver. 20.0 were used for analyses. Since the sample size was large in the present study, the significance level was set at *p <* .01. Correlation coefficients between SPS and age were significant (*p* < .001): *r* = -.118 (95% CI [-.161, -.075]) for the low sensory threshold, *r* = -.127 (95% CI [-.171, -.084]) for ease of excitation, and *r* = .140 (95% CI [.096, .183]) for the aesthetic sensitivity^1^.

**Fig. 1:**
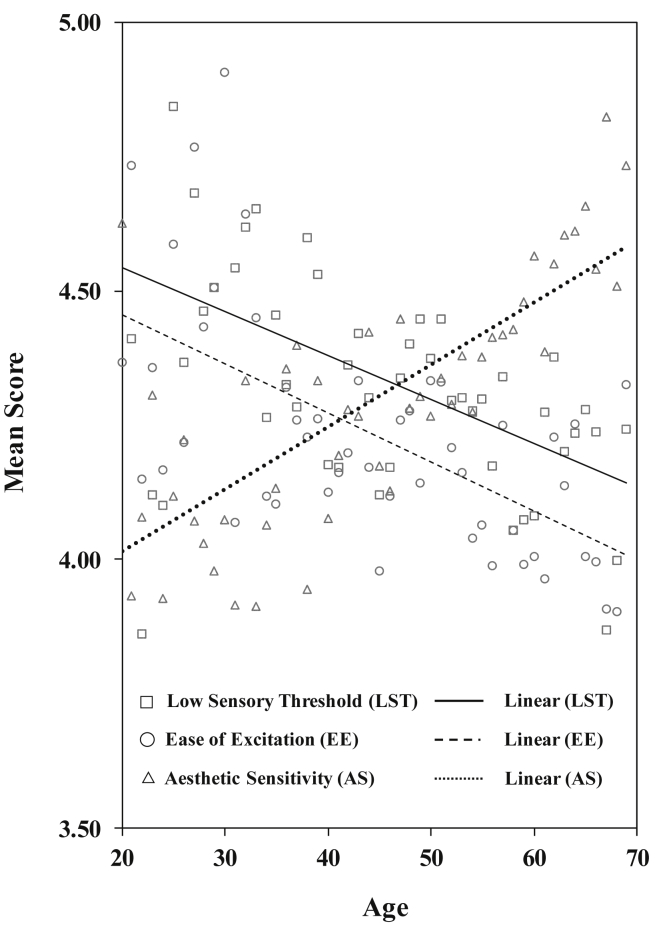
Estimated regression lines of the Japanese version of the 19-item Highly Sensitive Person Scale with significant age-related effects by controlling for sex.

This study conducted a hierarchical multiple regression analysis with sex (men = 0, women = 1), age, and age-squared as independent variables, and SPS as a dependent variable. Sex was entered in the first step, and age and age-squared in the second step. In the third step, interaction terms of sex and age and sex and age-squared were entered (Table 1). The results indicated that Δ*R*^2^ in the second step was significant for all dimensions of HSPS-J19. In the third step, Δ*R*^2^ values were not significant, indicating no significant interactive effects of sex and age on the dimensions. Low sensory threshold and ease of excitation indicated negative linear effects of age; aesthetic sensitivity indicated positive linear effects of age^2^. Fig. 1 shows the estimated marginal mean values and approximate lines of the three dimensions of SPS, controlling for sex.

**Table 1:** Hierarchical multiple regression analysis for sensory-processing sensitivity.

		Step 1			Step 2			Step 3		
		*β*	95% CI	*p*	*β*	95% CI	*p*	*β*	95% CI	*p*
Step 1										
Sex	L^a^	.117	[.073, .161]	<.001	.095	[.050, .140]	<.001	.093	[.049, .138]	<.001
	E^b^	.119	[.075, .163]	<.001	.096	[.051, .141]	<.001	.094	[.049, .139]	<.001
	A^c^	.093	[.049, .137]	<.001	.133	[.088, .177]	<.001	.132	[.088, .177]	<.001
Step 2										
Age	L				−.099	[−.144, −.053]	<.001	−.102	[−.149, −.056]	<.001
	E				−.103	[−.148, −.057]	<.001	−.104	[−.151, −.057]	<.001
	A				.178	[.133, .224]	<.001	.181	[.135, .228]	<.001
Age^2^	L				−.011	[−.055, .033]	.631	−.013	[−.059, .032]	.562
	E				.017	[−.027, .062]	.443	.012	[−.033, .058]	.594
	A				.046	[.001, .090]	.041	.036	[−.009, .081]	.117
Step 3										
Sex × Age	L							−.014	[−.060, .032]	.538
	E							−.023	[−.069, .023]	.330
	A							−.038	[−.083, .008]	.105
Sex × Age^2^	L							−.014	[−.060, .032]	.546
	E							−.003	[−.049, .043]	.909
	A							.022	[−.024, .067]	.347

*R*^2^	L	.014		<.001	.023		<.001	.023		<.001
	E	.014		<.001	.025		<.001	.026		<.001
	A	.009		<.001	.038		<.001	.040		<.001
Δ*R*^2^	L				.009		<.001	.000		.738
	E				.011		<.001	.000		.618
	A				.029		<.001	.002		.109

*Note*. ^a^L = Low Sensory Threshold, ^b^E = Ease of Excitation, ^c^A = Aesthetic Sensitivity, Age^2^ = Squared Term of Age.

## 4 Discussion

There were no significant interactive effects of sex and age, indicating that age-related changes in SPS do not differ by sex. Previously, differences in age effects between men and women were confirmed for neuroticism and openness (e.g., Soto et al., 2011). SPS showed an age-related change different from the Big Five personality traits, and it is possible that the change is the same in both sexes. However, the three dimensions of SPS showed correlations with sex in the first step, and some studies have confirmed sex differences in SPS among undergraduate students (e.g., Aron and Aron, 1997; Benham, 2006; Takahashi, 2016); we need to examine sex differences in a wide range of age groups.

The results indicate decreases with age for the dimensions of low sensory threshold and ease of excitation. Neuroticism, a concept similar to SPS, has been found to decrease linearly with age (Kawamoto et al., 2015; Soto et al., 2011). The present findings also showed that aesthetic sensitivity, considered a positive aspect of SPS, increases linearly with age. Openness, correlated with aesthetic sensitivity, has also been reported to increase linearly with age (Lehmann et al., 2013). Thus, the current findings confirm those in previous studies. However, the items on the scales of SPS and neuroticism are negatively worded, and the differences between the constructs have not been examined in detail. Further consideration will be needed to yield any findings about age and contracts differences between SPS and neuroticism. Furthermore, Terracciano et al. (2005) found that openness decreases after adulthood, whereas Kawamoto et al. (2015) suggest that openness has no correlations with age. Previous studies have also shown that age presents curvilinear relationships against neuroticism and openness (Lehmann et al., 2013; Terracciano et al., 2005). The effect size of the relationship between SPS and age obtained in the present study is small, and the measured age-related changes in these personality traits may differ by research method and across participants. In SPS as well, age-related changes are difficult to identify; it is necessary to pay sufficient attention to the interpretation of the results.

The interpretation of differences in age-related changes in each dimension of SPS was based on a consideration of the mutuality principle advocated by Caspi et al. (2005). Additionally, the previous study indicated that age-related changes in temperament traits change through development (Trouillet and Gana, 2008). Sensory functions such as visual and auditory functions have been found to decrease with age (Schumm et al., 2009). Physiological functions, such as nerve conduction velocity, basal metabolic rate, and pulmonary capacity, also decrease with age (Shock, 1971). SPS, an inherent individual difference in the processing of sensory information in the brain, may also change with age. According to the differential sustainability hypothesis advocated by Belsky (1997, 2005), there is a sensitive group for both negative and positive stimuli. High-sensitivity groups are prone to depression and anxiety in stressful environments; in contrast, they may develop more healthily than low sensitive groups in environments where their traits are understood and supported. That is to say, the degrees to which people are influenced by the quality of experience and environment differ among individuals, and responds positively to not only negative effects but also positive effects. There is a possibility that SPS changes over time and may typically be affected by the tasks performed and events experienced at each developmental stage, and there is large variation among individuals (Belsky, 1997, 2005).

A limitation of this study is its examination of simulated developmental processes using cross-sectional data, which may not always accurately reflect actual developmental processes. In the present study, we have focused on the three dimensions of SPS. However, further research will be needed to examine age-related changes of the high-sensitive groups. Following the research of Lionetti et al. (2018), it is necessary to extract highly sensitive groups by latent class analysis and conduct longitudinal studies with a specified group. Although the present study conducted a survey of Japanese people, an international comparison between those who belong to various cultures would be desirable in reference to the cultural differences of SPS. Furthermore, future studies should include people in their teens and those above 70 years of age to examine developmental processes in greater detail.

## Declarations

### Author contribution statement

Yuki Ueno: Conceived and designed the experiments; Performed the experiments; Analyzed and interpreted the data; Contributed reagents, materials, analysis tools or data; Wrote the paper.

Aki Takahashi, Atsushi Oshio: Conceived and designed the experiments; Analyzed and interpreted the data; Wrote the paper.

### Funding statement

This project was supported by JSPS KAKENHI 25380893, Kwansei Gakuin University Joint Research Grant(B), JSPS KAKENHI 16J00972, JSPS KAKENHI 16J07940.

### Competing interest statement

The authors declare no conflict of interest.

### Additional information

No additional information is available for this paper.

## Footnotes

1) The score of the low sensory threshold showed correlations with ease of excitation (*r* = .755, 95% CI [.736, .774], *p* < .001), aesthetic sensitivity (*r* = -.045, 95% CI [-.089, -.001], *p* = .045), and correlation among the ease of excitation and aesthetic sensitivity (*r* = -.065, 95% CI [-.109, -.021], *p* = .004). The total score of the HSPS-J19 showed significant negative correlations with age (*r* = -.092, 95% CI [-.135, -.048], *p* < .001).

2) After controlling for sex in the first step, the total score of the HSPS-J19 indicated no significant effect of age (*β* = -.058, 95% CI [-.104, -.013], *p* = .012).
